# Exogenous L-Serine Alleviates *Pasteurella multocida*-Induced Inflammation by Reprogramming the Transcription and Metabolism of Macrophages

**DOI:** 10.3390/vetsci12030254

**Published:** 2025-03-07

**Authors:** Fang He, Zhengchun Lang, Yanlan Huang, Yangyang Qiu, Pan Xiong, Nengzhang Li, Guangfu Zhao, Yuanyi Peng

**Affiliations:** College of Veterinary Medicine, Southwest University, Chongqing 400715, China; hefang2017@sina.com (F.H.); swulzc@163.com (Z.L.); 15177594549@163.com (Y.H.); 17092156425@163.com (Y.Q.); x1741852320@163.com (P.X.); lich2001020@163.com (N.L.)

**Keywords:** L-serine, *Pasteurella multocida*, macrophage, inflammasome, metabolic reprogramming

## Abstract

*P. multocida* is notorious for inducing excessive inflammation with high lethality in multiple animals, such as cattle, pigs, and chickens. Our previous study revealed that L-serine was decreased in the lungs of mice infected with *P. multocida* capsular type A strain CQ2 (PmCQ2), and 2 mg/kg of L-serine could alleviate PmCQ2-induced lung inflammation in vivo, which may largely depend on macrophages. However, the underlying intrinsic alterations remain unknown. Here, we demonstrated that 10 mM of L-serine significantly inhibited the release of inflammatory cytokines (e.g., IL-1β and TNF-α) by blocking inflammasome activation (including NALP1, NLRP3, NLRC4, AIM2, and Caspase-1) in PmCQ2-infected macrophages. Furthermore, the results of RNA-seq and metabonomics revealed that exogenous L-serine supplementation substantially reprogrammed macrophage transcription and metabolism. Mechanically, L-serine reduced inflammatory responses via the inhibition of glycolysis in macrophages based on a seahorse assay. Together, these findings characterize the intrinsic molecular alterations in activated macrophages and provide new targets for modulating *P. multocida* infection-induced macrophage inflammation.

## Simple Summary

Pathogenic bacterial infection is the principal factor leading to respiratory diseases such as bovine pneumonia, which has caused huge economic losses to the livestock industry. *Pasteurella multocida* (*P. multocida*) is the most common pathogen resulting in bovine pneumonia. Currently, the disease is mainly controlled through vaccination and antibiotics, but the results are not satisfactory. Therefore, the search for efficient and safe natural substances is of great significance for the prevention and control of *P. multocida* infection. Research indicates that amino acids possess diverse physiological functions, encompassing immune regulation, anti-inflammation, and anti-oxidation. Based on the previous studies, we investigated the role and mechanism of L-serine in macrophages infected with *P. multocida*. This offers a novel strategy for the prevention and control of bovine pneumonia.

## 1. Introduction

Macrophages are key innate immune cells that are highly diverse and plastic in response to various stimuli, regulating a broad range of inflammatory processes (including microbial infection-induced inflammation) [1,2]. For example, we previously reported that *P. multocida* [an opportunistic pathogenic bacterium capable of causing infections in poultry, livestock, and even humans] induces macrophage-mediated inflammatory responses (e.g., caspase-1 activation and IL-1β secretion) in mice [3,4,5]. Additionally, metabolic/cellular pathways and/or metabolites are also responsible for the growth, survival, and effector functions of macrophages [6,7]. More importantly, bacterial infections can influence the function and activation of macrophages and reprogram intracellular metabolism (including amino acid metabolism) [8,9].

In addition to its nutritional effects, increasing evidence has demonstrated that L-serine, a nonessential amino acid, is deeply involved in cell proliferation, the stress response, inflammation [10,11], and the treatment of diseases [12,13]. We have shown that 1.2 mM of L-serine can regulate IL-1β production in macrophages through mTOR signaling [12]. Moreover, Rodriguiz et al. reported that 400 μM of L-serine supports macrophage IL-1β production via the serine–glycine–glutathione axis [14]. Additionally, we found that the *P. multocida* serotype A CQ2 strain (PmCQ2) infection reduces L-serine levels in the lungs of mice and that 2 mg/kg of L-serine supplementation alleviated lung inflammation in mice, which largely depends on macrophages [15]. However, whether high exogenous levels of L-serine can directly affect macrophage function is unclear, and the intrinsic molecular alterations in activated macrophages exposed to L-serine remain to be characterized.

Consequently, this research is designed to explore the concrete role and inherent molecular mechanism of L-serine in macrophages infected with *P. multocida*, in the hope of offering a novel strategy for the prevention and control of bovine pneumonia.

## 2. Materials and Methods

**Strains and culture conditions**: The highly virulent bovine *P. multocida* capsular type A CQ2 (PmCQ2) was stored in our lab with defined genome (GenBank accession number: No. CP033599), which was isolated from the lungs of a calf with pneumonia in Chongqing, China [16]. PmCQ2 was streaked on Martin agar plates (Qingdao Hope Biol-Technology Co., Ltd., Qingdao, China) at 37 °C for 24 h, after which a single colony was transferred to 5 mL Martin broth and cultured for 12 h at 37 °C with shaking (200 r/min) [17].

**Experimental animals and ethics statement**: Female C57BL/6 mice aged 6–8 weeks were procured from Hunan SJA Laboratory Animal Co., Ltd. (Changsha, China). Upon arrival, the animals were housed in individually ventilated cages under specific pathogen-free conditions. The environmental parameters were maintained at a temperature of 20–30 °C, relative humidity of 50–60%, and a light cycle of 12 h light/dark. The acclimation period lasted four days prior to the commencement of the experiment. All the animal experiments were approved by the Chongqing Laboratory Animal Management Committee [License No: SYXK (Yu) XK2019-0003] and were performed in strict accordance with the guidelines of the Basel Declaration and the recommendations of the Laboratory Animal Ethical Commission of Southwest University to minimize animal suffering.

**Antibodies**: mTOR (20657-1-AP, 1 500, Proteintech, Rosemont, IL, USA), p-mTOR (ab137133, 1:1000, Abcam, Cambridge, UK), HIF-1α (14179s, 1:1000, CST, America), IKKα/β (SC-7607, 1:200, Santa Cruz, Texas, USA), p-IKKα/β (SC-21660, 1:1000, Santa Cruz, TX, USA), p-IKBα/β (9246s, 1:1000, CST, MA, USA), IKBα/β (51066-1-AP, 1:500, Proteintech, Rosemont, IL, USA), p65 (ab31481, 1:500, Abcam, Cambridge, UK), p-p65 (bs-0982r, 1:500, Bioss, Beijing, China), NALP1 (PA5-17275, 1:1000, Invitrogen Antibodies, Carlsbad, CA, USA), NLRP3 (ab214185, 1:200, Abcam, Cambridge, UK), NLRC4 (bs-20016R, 1:1000, Bioss, Beijing, China), AIM2 (ab180665, 1:1000, Abcam, Cambridge, UK), IL-1β (12426s, 1:1000, CST, MA, USA), and Caspase-1 (ab179515, 1:1000, Abcam, Cambridge, UK).

**Murine primary peritoneal exudate macrophage** (**PEM**) **isolation and cytotoxicity tests**: PEMs were isolated from mice as previously described [18]. Briefly, three days after an injection of 4% mercaptoacetic acid (Eiken, Tokyo, Japan), mice were euthanized by intraperitoneal injection of 100 μL of pentobarbital sodium (1.5%). Peritoneal exudate macrophages (PEMs) were collected from the peritoneal cavity with 100 μL of cold RPMI 1640 medium (Gibco, Waltham, MA, USA) containing 0.286 mM of L-serine. Subsequently, the isolated PEMs were cultured in RPMI 1640 medium (Gibco, Waltham, MA, USA) supplemented with 10% fetal bovine serum and 1% penicillin/streptomycin at 37 °C and 5% CO_2_ for 4 h. Then, the cells were washed with PBS to remove non-adherent cells. Adherent cells were cultured in RPMI 1640 medium supplemented with L-serine (1 mM, 5 mM or 10 mM) for 2 h, followed by challenge with 2.2 × 10^5^ CFU Pasteurella multocida for 8 h and/or 12 h. The supernatants were collected for determination of cytokines, LDH (TaKaRa, Kusatsu, Japan), and NO (Beyotime, Shanghai, China), and the cells were collected for further processing.

**Cell apoptosis analysis**: To assess cell apoptosis, Annexin V/PI staining was employed. Specifically, following the designated treatments, 1 × 10^7^ PEMs (including both floating and adherent cells) were gently harvested and stained using a FITC Annexin V/PI apoptosis detection kit (BD Biosciences, Milpitas, CA, USA) according to the manufacturer’s instructions. The analysis of apoptosis was conducted using flow cytometry (FACSCalibur, BD Biosciences, Milpitas, CA, USA).

**Quantitative real-time-PCR** (**qRT-PCR**): The total RNA of the macrophages was extracted using an RNAprep Pure Cell Kit (TIANGEN, Beijing, China) involving a gDNA elimination step. cDNAs were synthesized with a First-Strand cDNA Synthesis Kit (US Everbright Inc., R2028, Suzhou, China), and qRT-PCR was performed according to previous methods via a CFX96 instrument (Bio-Rad, Hercules, CA, USA) [19]. Each target gene was individually normalized to the reference gene (β-actin) via the 2^−∆∆Ct^ method. The primer sequences are listed in Supplementary Materials Table S1.

**Enzyme-linked immunosorbent assay** (**ELISA**): The concentration of inflammatory cytokines (IL-1β, TNF-α, IL-6, and IL-12) in the cell culture supernatants were analyzed with mouse ELISA kits (Invitrogen, Carlsbad, CA, USA) in accordance with the manufacturer’s protocol.

**Immunoblotting analysis of macrophages**: The Western blot analysis was performed as follows. Briefly, 2.5 × 10^6^ PEMs (n = 4) were lysed in 180 μL of ice-cold RIPA buffer for 10 min. The protein concentration of the supernatant was measured with a bicinchoninic acid (BCA) protein assay kit (Beyotime, Shanghai, China). A total of 10 μg of protein was separated via 10% SDS–PAGE electrophoresis. Proteins were transferred onto a PVDF membrane (Bio-Rad, Hercules, CA, USA) and blocked with 5% BSA for 1.5 h. The membranes were incubated with primary antibodies overnight at 4 °C. After that, membranes were incubated with corresponding Horseradish peroxidase (HRP)—conjugated secondary antibodies (anti-rabbit 1:6000, Abcam, Cambridge, United Kingdom; anti-mouse 1:5000, Proteintech, Rosemont, IL, USA; anti-goat 1:30,000, Santa Cruz, TX, USA) for 90 min at 37 °C. Finally, the membranes were observed through using AlphaImager 2200 software (version 4.0, Alpha Innotech Corporation, San Leandro, CA, USA). The signal intensity was quantified and normalized to the protein abundance of actin.

**Transcriptomic analysis**: A total of 2 × 10^6^ PEM samples were sent to NovoGene (Beijing, China) for transcriptome sequencing and analysis. The specific methods used were previously described [20]; briefly, total RNA was extracted via TRIzol reagent (Invitrogen Life Technologies, Carlsbad, CA, USA) following the manufacturer’s protocol. Next, rRNA was removed via a Ribo-Zero rRNA Removal Kit (Illumina, San Diego, CA, USA). Then, 1 μg of each RNA sample was used to construct a strand-specific cDNA library according to the recommendations of the Illumina TruSeq Stranded Kit. The cDNA library size was analyzed via an Agilent 2100 Bioanalyzer (Santa Clara, CA, USA), and the effective concentration was determined via qPCR (StepOnePlus Real-Time PCR Systems, Thermo Scientific, Waltham, MA, USA). Finally, the samples were sequenced on the Illumina sequencing platform (HiSeq 4000), and 150 bp paired-end reads were generated. The reads were aligned [21], and reads were discarded with an average base quality lower than Q20. The clean reads were mapped to the genome [22] and to the reference gene set [23], after which RSEM software (version 1.3.1) [24] was used to calculate gene expression levels. Differential expression analysis was performed using estimate size factors via the DESeq (version 1.18.0) R package. *p* < 0.05 and |log2fold change| ≥ 1 were considered the thresholds for DEG identification. All the data in this study were deposited in the NCBI Sequence Read Archive (SRA) database, and the accession number is PRJNA527189.

**Metabolite analysis**: 1 × 10^7^ PEMs were quickly collected and frozen in liquid nitrogen, after which the cell samples were sent to Shanghai Baiqu Biological Technology Co., Ltd. (Shanghai, China, for metabolite detection and analysis via gas chromatography–mass spectrometry (GC-TOF-MS). The cells were resuspended into PBS and centrifuged at 8000 rpm for 10 min, and 1 mL of methanol was added to sonicate for 10 min to disrupt the cells. The PEM supernatant was passed through a 0.22 µm water phase membrane filter, and, then, 5 µL of the sample was injected to Hitachi L8900 (Tokyo, Japan) high-efficiency automatic amino acid analyzer for quantitative analysis.

**Seahorse assay**: Seahorse XF24 Extracellular Flux Analyzer (Agilent Technologies, Santa Clara, CA, USA) was used to analyze the changes in the OCR (oxygen consumption rate) and ECAR (extracellular acidification) of PEMs (2 × 10^5^ cells). OCR assays were performed with 2 mM of glutamine and 10 mM of glucose and adjusted to pH 7.4. Then, the following compounds were added in sequence: 100 μM of oligomycin, 100 μM of carbonyl cyanide 4-(trifluoromethoxy) phenylhydrazone (FCCP), and 50 μM of rotenone plus antimycin A. ECAR assays were performed with 1 mM of glucose, 1 mM of oligomycin, and 1 mM of 2-deoxy-glucose (2-DG).

**Statistical analyses**: The data shown are the means ± SDs. Data between two groups were analyzed by an unpaired *t* test (Prism 8.0) if the data were Gaussianly distributed and had equal variance, by unpaired *t* test with Welch’s correction (Prism 8.0) if the data were Gaussianly distributed but with unequal variance, or by nonparametric test (Mann–Whitney U test, Prism 8.0) if the data were not normally distributed. Data from more than two groups were analyzed by one-way ANOVA followed by Dunnett’s multiple comparisons test (Prism 8.0) if the data were Gaussianly distributed and had equal variance or analyzed by Kruskal–Wallis test followed by Dunn’s multiple comparisons test (Prism 8.0) if the data were not normally distributed. The Gaussian distribution of the data was analyzed via the D’Agostino–Pearson omnibus normality test (Prism 8.0) and the Kolmogorov–Smirnov test (Prism 8.0). The variance of the data was analyzed via the Brown–Forsythe test (Prism 8.0). Differences with *p* < 0.05 were considered significant.

## 3. Results

**Figure 1:**
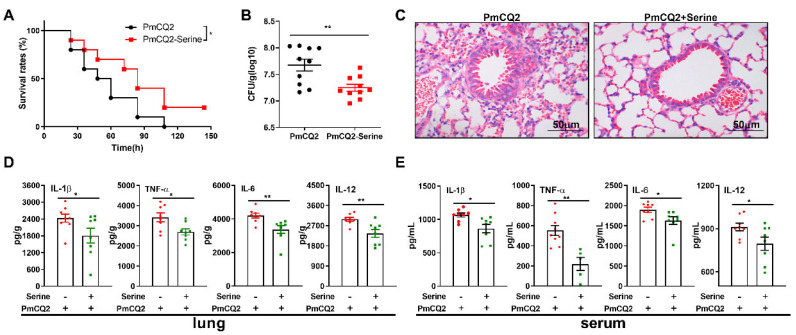
Intragastric supplementation with exogenous L-serine is capable of effectively resisting *P. multocida* infection: (**A**) L-serine improves the survival rate of the mice (n = 10). (**B**) L-serine markedly decreases the bacterial burden in the lungs of mice infected with *P. multocida* for 24 h (n = 10). (**C**) H&E staining is used to analyze inflammatory lesions in the lungs of mice after 24 h of infection with *P. multocida* (n = 6). (**D**,**E**) L-serine significantly inhibits the secretion of IL-1β, TNF-α, IL-6, and IL-12 in the lungs and serum of mice following 24 h of infection with *P. multocida* (n = 8). The data are representative of two independent experiments and are analyzed via unpaired test or Mann–Whitney test; the results are expressed as the means ± SD. PmCQ2: *P. multocida*. * *p* < 0.05, ** *p* < 0.01.

**Exogenous L-serine provides effective protection against *P****. **multocida*****infection**. Consistent with our previous study [15], gavage with serine also significantly enhanced the survival rate of *P. multocida*—infected mice (Figure 1A), reduced bacterial colonization in the lungs (Figure 1B), attenuated pulmonary pathological damage (Figure 1C), and diminished the secretion of inflammatory cytokines (IL-1β, TNF-α, IL-6, and IL-12) in both lung tissue and serum (Figure 1D,E). These results demonstrate that serine is indeed effective in resisting *P. multocida* infection. However, the underlying intrinsic alterations remain unknown.

**Figure 2:**
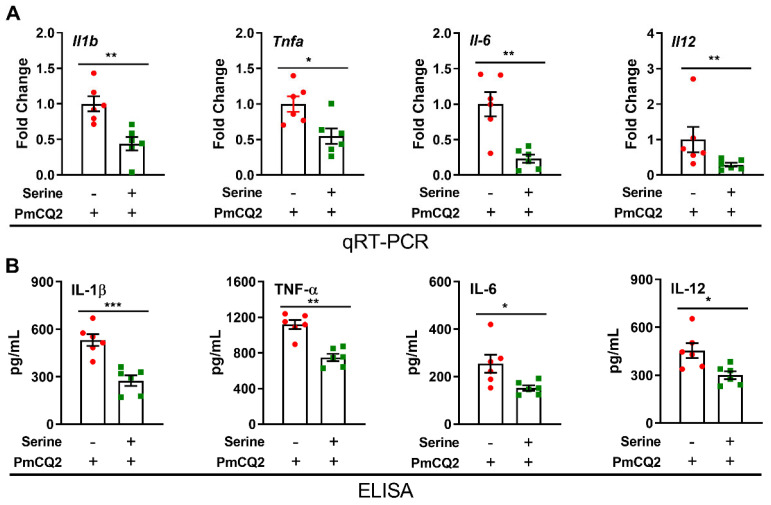
L-serine (10 mM) inhibits the production of inflammatory cytokines in proinflammatory macrophages activated with *P. multocida*: (**A**,**B**) L-serine (10 mM) inhibits the mRNA (n = 6) expression and production (n = 8) of IL-1β, TNF-α, IFN-γ and IL-17 in macrophages infected by *P. multocida* (Mann–Whitney U test or unpaired Student’s *t*-test). The samples are collected at 12 h post-treatment. The data are representative of three independent experiments, and all data are expressed as means ± SD. * *p* < 0.05, ** *p* < 0.01, *** *p* < 0.001.

**Exogenous L**-**serine inhibits the production of inflammatory cytokines in proinflammatory macrophages**. We first investigated the direct effects of L-serine (1 mM, 5 mM, and 10 mM) on the viability of macrophages, and we found that L-serine did not exhibit significant effects on cell viability (Supplementary Materials Figure S1A) or the NO production from macrophages (Supplementary Materials Figure S1B). Considering that proinflammatory macrophages determine the orchestration of immune responses [25], we asked whether exogenous L-serine could influence the physiological state of proinflammatory macrophages. The results revealed that 10 mM of L-serine significantly inhibited the apoptosis of activated macrophages (Supplementary Materials Figure S1C), suggesting that a high dose of exogenous L-serine may play a beneficial role in regulating macrophage function. Proinflammatory macrophages are characterized by the secretion of large amounts of mediators, including IL-1β and TNF-α [26]. Here, we found that 10 mM of L-serine significantly reduced the expression of inflammatory cytokines (mainly IL-1β and TNF-α) in *P. multocida*—infected macrophages (the most significant changes were at 12 h post infection) (Figure 2A,B and Supplementary Materials Figure S1D,E). Overall, L-serine is critical for modulating macrophage function, particularly in determining IL-1β production in proinflammatory macrophages.

**Figure 3:**
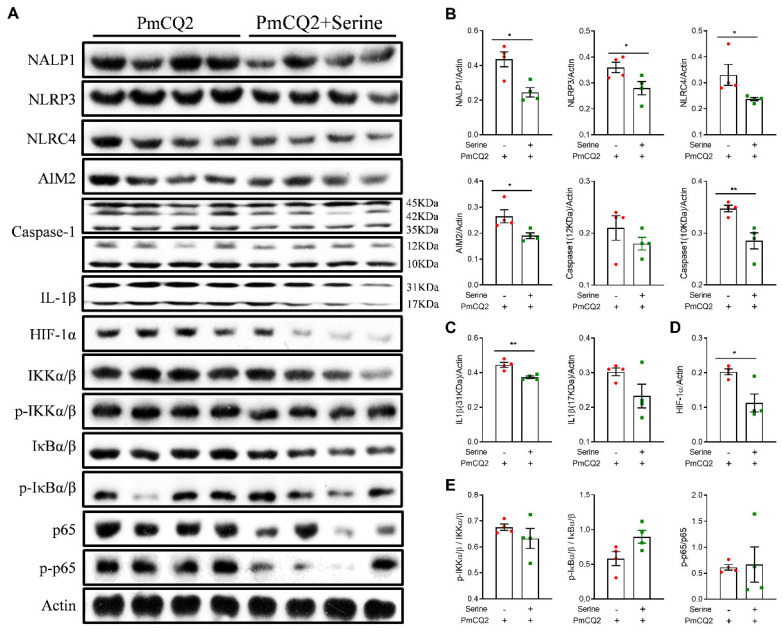
L-serine (10 mM) blocks inflammasome activation in proinflammatory macrophages: (**A**) The protein abundance of NALP1, NLRP3, NLRC4, AIM2, Caspase-1, IL-1β, HIF-1α, IKKα/β, p-IKKα/β, IkBα/β, p-IkBα/β, p65, and p-p65 in macrophages by Western blotting. (**B**) The activation of inflammasomes, including NALP1, NLRP3, NLRC4, AIM2, and Caspase1 (10 kDa) in macrophages (n = 4, Mann–Whitney U tests). (**C**) The statistical analysis of IL-1β levels in macrophages (n = 4, Mann–Whitney U tests). (**D**) The protein abundance of HIF-1α in macrophages (n = 4, Mann–Whitney U tests). (**E**) The activation of IKK/NF-κB in macrophages (n = 4, Mann–Whitney U tests). The samples are collected at 12 h post treatment. The data are representative of two independent experiments and are expressed as means ± SD. * *p* < 0.05, ** *p* < 0.01.

**Exogenous L-serine blocks inflammasome activation and reduces HIF-1α expression in proinflammatory macrophages**. Because the activation of cellular signaling pathways (e.g., NF-κB and inflammasomes) is responsible for the function of proinflammatory macrophages [27,28,29], we performed Western blot to analyze the abundance of proteins in these pathways, and we found that 10 mM of L-serine substantially decreased the protein expression of NALP1, AIM2, NLRP3, NLRC4, and cleaved-caspase-1 (Figure 3A,B), which are vital platforms for production of the key inflammatory mediator (IL-1β) [30]. Furthermore, 10 mM of L-serine highly reduced the protein expression of IL-1β (Figure 3A,C), which is consistent with the results shown in Figure 2. However, L-serine had little effect on the activation of NF-κB signaling (Figure 3A,D,E), which significantly inhibited the activation of HIF-1α. In summary, these data demonstrate that 10 mM of L-serine inactivates the inflammasome and reduces HIF-1α expression in PmCQ2-infected macrophages.

**Figure 4:**
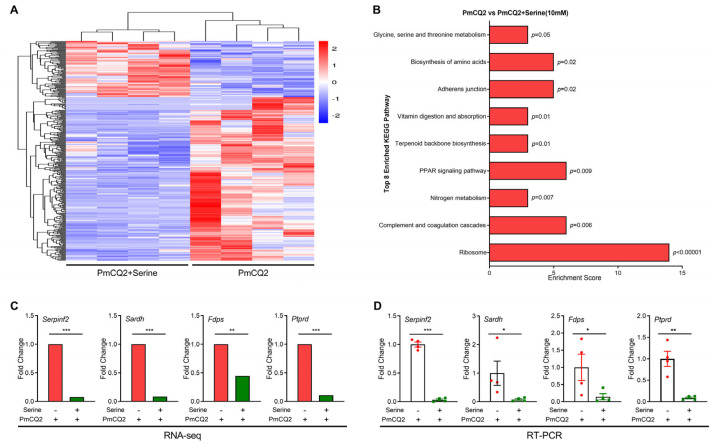
L-serine (10 mM) alters the transcriptomic profile of proinflammatory macrophages: (**A**) The heat-map clustering of DEGs in macrophages via transcriptome sequencing (n = 4). (**B**) The eight top KEGG pathways in macrophages after a 10 mM L-serine treatment. (**C**,**D**) DEGs identified via RNA-seq (**C**) and qRT-PCR (**D**) (n = 3, unpaired two-tailed Student’s *t*-test). The samples are collected at 12 h post treatment. Data are representative of two independent experiments, and all the data are expressed as means ± SD. * *p* < 0.05, ** *p* < 0.01, *** *p* < 0.001.

**Exogenous L-serine alters the transcriptomic profile of proinflammatory macrophages**. To explore influence of 10 mM of L-serine on proinflammatory macrophage function, the transcriptome sequencing of proinflammatory macrophages was performed. L-serine (10 mM) significantly shaped the transcriptomic profile of activated macrophages (Figure 4A and Table 1), with 348 differentially expressed genes (DEGs) detected (FC > 2, *p* < 0.05). Among these genes, 88 genes were upregulated, and 260 genes were downregulated (Supplementary Materials Figure S2A). Functional enrichment analysis showed that the DEGs were enriched mostly in biological processes. KEGG enrichment revealed that total 70 KEGG pathways were enriched (input number > 3), and 19 KEGG pathways showed significant differences (input number > 3, *p* < 0.05) (Supplementary Materials Figure S2B–E and Supplementary Materials Table S2). The enrichment score of inflammation and metabolism-related pathways has been significantly changed, including ribosomes, complement and coagulation cascades, nitrogen metabolism, the PPAR signaling pathway, terpenoid backbone biosynthesis, vitamin digestion and absorption, adherence junction, the biosynthesis of amino acids, and glycine, serine and threonine metabolism (Figure 4B). To validate the results of RNA-seq analysis, four DEGs were randomly chosen for qPCR analysis, and the results were similar to those obtained from the RNA-seq method (Figure 4C,D). In summary, 10 mM of L-serine altered the transcriptomic profile of proinflammatory macrophages, especially substance biosynthesis and metabolism (e.g., amino acids).

**Figure 5:**
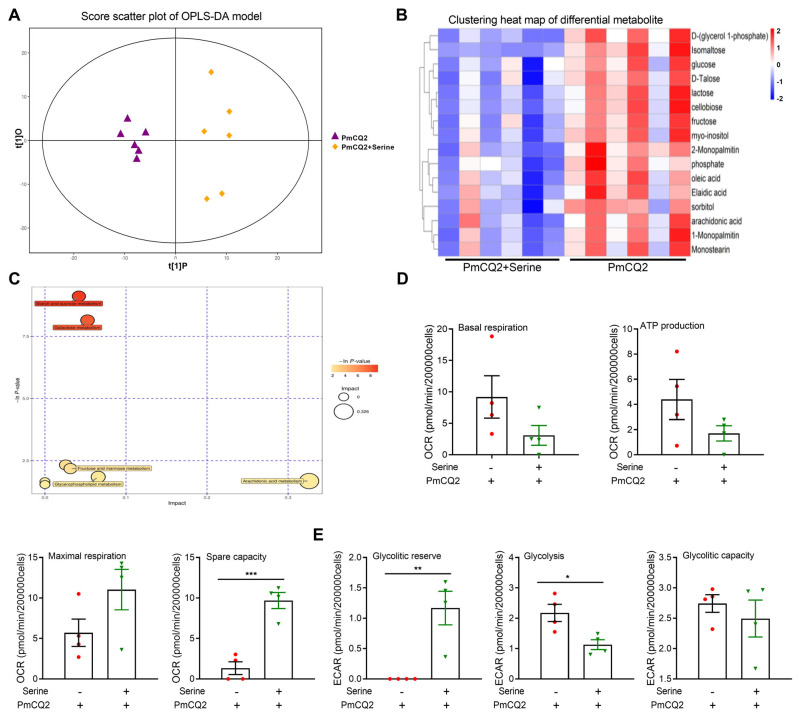
L-serine (10 mM) shapes the metabolic profile of proinflammatory macrophages: (**A**) OPLS-DA score plot. (**B**) The clustering heatmap of significantly different metabolites via metabolomics (n = 6). (**C**) Significant metabolic pathways in macrophages after a 10 mM L-serine treatment. (**D**,**E**) Effects of 10 mM of L-serine on oxidative phosphorylation and (**D**,**E**) glycolysis in macrophages analyzed via seahorse analysis (n = 4, Mann–Whitney U tests). The samples are collected at 12 h post-treatment. (**D**,**E**) Data are representative of three independent experiments and expressed as means ± SD. * *p* < 0.05, ** *p* < 0.01, *** *p* < 0.001.

**Exogenous L-serine shapes the metabolic profile of proinflammatory macrophages**. The cellular metabolism is strongly associated with functional output in the immune system [6]. Therefore, we performed metabolomics to identify the differentially abundant metabolites in PmCQ2 vs. PmCQ2 + serine macrophages. The results were reliable according to the metabolomic data (Figure 5A and Supplementary Materials Figure S3A,B). Treatment with 10 mM of L-serine significantly altered various metabolites in activated macrophages, including lower levels of glucose, isomaltose, lactose, cellobiose, fructose, and myo-inositol (Figure 5B,C), which indicated that supplementation with 10 mM of L-serine influenced glycolysis, which is needed for proinflammatory macrophages. Subsequently, we assessed cellular bioenergetics and found that a 10 mM L-serine supplementation inhibited glycolysis in activated macrophages but had little effect on oxidative phosphorylation (Figure 5D,E). Taken together, these findings indicate that 10 mM of L-serine shapes the metabolic profile and restricts glycolysis in proinflammatory macrophages.

## 4. Discussion

The *P. multocida* serotype A PmCQ2 mainly causes pneumonia, resulting in considerable economic losses to the breeding industry [31,32,33]. Our previous study demonstrated that 2 mg/kg of L-serine reduced macrophage- and neutrophil-mediated lung inflammation during PmCQ2 infection but did not affect macrophage phagocytosis [15]. Previously, we reported that a low dose of exogenous (1.2 mM) L-serine supported IL-1β production in macrophages through mTOR signaling [12]. Moreover, another study suggested that 400 μM of L-serine highly regulates macrophage IL-1β production through the serine–glycine–glutathione axis [14] and that 400 μM of L-serine orchestrates macrophage polarization by regulating the IGF1–p38 axis [34]. Interestingly, we also found that a high concentration of L-serine (10 mM) inhibited the apoptosis and production of inflammatory cytokines in macrophages dependent on inflammasomes (NALP1, AIM2, NLRP3, NLRC4 and Caspase-1) in this study. Thus, exploring the mechanisms by which L-serine (different concentrations) regulates macrophage biology is interesting.

On the basis of the expression of markers, activated macrophages can be classified as classically activated (M1) macrophages or tissue-repaired (M2) macrophages [7]. IKK/NF-κB signaling pathways are involved in the regulation of inflammatory responses [28], and NF-κB is a key transcriptional regulator of M1 macrophages [35]. However, we found that 10 mM of L-serine had little effect on the activation of the IKK/NF-κB pathway in PmCQ2-infected macrophages. HIF-1α is an important molecule that regulates the function of macrophages under hypoxia and can promote anaerobic glycolysis and phosphate pentose pathway metabolic reprogramming, thereby affecting macrophage polarization toward the M1 type [36]. Here, we found that 10 mM of L-serine significantly inhibited the protein abundance of HIF-1α, indicating that serine regulates glycolytic metabolic reprogramming through HIF-1α, thereby affecting macrophage polarization. Notably, inflammasomes, including NALP1, AIM2, NLRP3, NLRC4, and Caspase-1, are also essential for regulating the inflammatory responses in macrophages [27]. Mechanistically, inflammasomes activate the proinflammatory protease Caspase-1 to promote the maturation and secretion of IL-1β and induce apoptosis [29]. In this study, 10 mM of L-serine inhibited the activation of inflammasomes (NALP1, AIM2, NLRP3, NLRC4 and Caspase-1) in macrophages, which is consistent with the observation that L-serine markedly reduced inflammation in PmCQ2-infeced mice. However, it is interesting to explore the underlying mechanisms.

Interestingly, we also found that 10 mM of L-serine significantly blocked the glycolysis of PmCQ2-infected macrophages without changing the level of oxidative phosphorylation. M1 macrophages are highly dependent on glycolysis because of their high energy demands and drastic inflammatory reactions [6]. Inhibiting glycolysis in activated macrophages significantly restricts inflammatory reactions [6,37]. Thus, the underlying mechanism by which L-serine restricts the macrophage inflammation response may depend on the inhibition of glycolysis by suppressing the activity or expression of key glycolytic enzymes. However, the potential mechanisms that are possible still require further investigation.

Additionally, the results of the transcriptome sequencing of macrophages revealed that many genes, such as the peroxisome proliferator activated receptor (PPAR), were significantly upregulated or downregulated after a 10 mM L-serine treatment. The PPAR is a ligand-dependent transcript that regulates glucose and lipid metabolism and is widely expressed in human and mouse macrophages to inhibit the expression of proinflammatory genes [38]. These findings suggest that 10 mM of L-serine strongly influences the metabolic and transcriptional reprogramming in macrophages. Similarly, there were great alterations in the levels of intracellular metabolites in 10 mM L-serine-treated M1 macrophages; however, how 10 mM of L-serine influences the levels of those metabolites and whether it reduces IL-1β and TNF-α production in M1 macrophages via these metabolite-mediated mechanisms still need a comprehensive investigation.

## 5. Conclusions

We demonstrated that L-serine supplementation can tailor the production of inflammatory cytokines (e.g., IL-1β and TNF-α) in PmCQ2-infected macrophages by blocking inflammasome activation (including NALP1, AIM2, NLRP3, NLRC4, and Caspase-1). Additionally, L-serine supplementation substantially reprograms macrophage transcription and metabolism according to the results of RNA-seq and metabonomics. A seahorse assay revealed that L-serine can reduce inflammatory responses via inhibition of the glycolysis process in macrophages. Thus, these findings provide new targets for modulating macrophage-dependent inflammation.

## Figures and Tables

**Table 1:** Statistics and quality assessment of transcriptome sequencing results.

Sample Name	Raw Reads	Clean Reads	Clean Bases	Q20 (%)	Q30 (%)	GC Content (%)	Uniquely Mapped
Control1	5,359,8470	52,766,552	7.91G	97.2	92.73	51.78	45,933,660 (87.05%)
Control2	56,675,122	55,129,440	8.27G	98	94.79	52.38	46,941,892 (85.15%)
Control3	58,821,956	56,695,884	8.5G	98	94.62	52.18	48,879,328 (86.21%)
Control4	49,940,592	49,166,168	7.37G	97.4	92.92	51.83	42,542,372 (86.53%)
Ser9	49,807,792	48,215,352	7.23G	98	94.66	52.62	41,203,396 (85.46%)
Ser10	54,377,336	52,357,056	7.85G	98	94.77	52.02	45,177,015 (86.29%)
Ser11	55,734,868	54,474,590	8.17G	98.1	94.86	52.15	47,368,843 (86.96%)
Ser12	5,6845,112	55,463,248	8.32G	98.1	94.78	52.23	47,843,443 (86.26%)

## Data Availability

All the data in this study were deposited in the NCBI Sequence Read Archive (SRA) database, and the accession number is PRJNA527189.

## Supplementary Materials

The following supporting information can be downloaded at: https://www.mdpi.com/article/10.3390/vetsci12030254/s1, Figure S1: The effect of L-serine on the function of macrophage; Figure S2: The results of transcriptome analysis in L-serine/Control; Figure S3: The results of metabolome analysis in L-serine/Control; Table S1: Primers used in qRT-PCR analysis; Table S2: PmCQ2+Ser vs PmCQ2_KEGG_pathway. The original Western blots were published as Supplementary Materials.
