# Supplementary material for: Exogenous L-Serine Alleviates Pasteurella multocida-Induced Inflammation by Reprogramming the Transcription and Metabolism of Macrophages
Source: Vet Sci. 2025 Mar 7;12(3):254. doi: 10.3390/vetsci12030254 (PMC11945856; doi:10.3390/vetsci12030254)
Supplement: Supplementary file 1 [file vetsci-12-00254-s001.zip › Supplementary material.pdf]

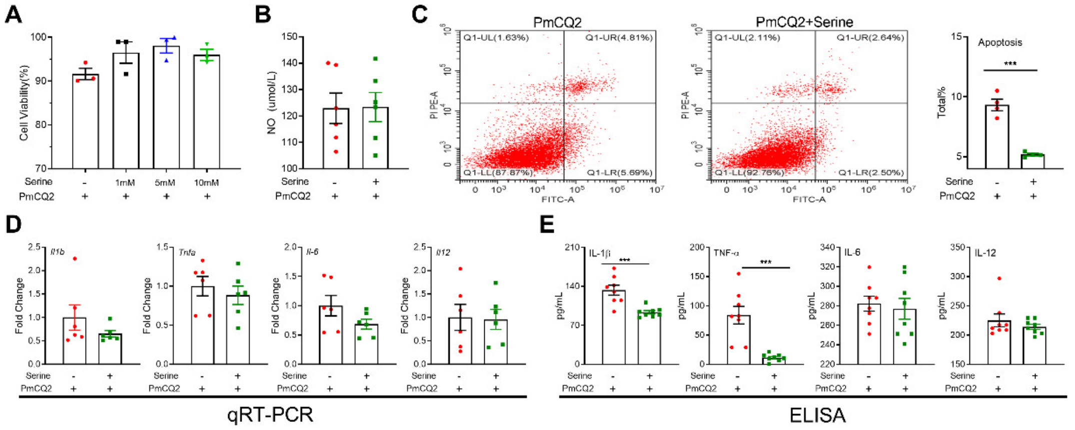

Supplementary Figure S1. The effect of L-serine on the function of macrophages. A: L-serine (1 mM, 5 mM, 10 mM) does not affect the cell viability of pro-inflammatory macrophages (n=3, One way ANOVA). **B:** 10 mM L-serine does not affect the production of NO (n=3, unpaired two-tailed Student's t-test). **C:** 10 mM L-serine decreased the apoptosis of macrophages activated by IFN-γ plus LPS (n=4, unpaired two-tailed Student's t-test). **D&E:** 10 mM L-serine inhibits the mRNA expression (n=6) and protein production (n=8) of IL-1β, TNF-α, IL-6 and IL-12 in macrophages at 8 h post infected by *P. multocida* (unpaired two-tailed Student's t-test, Mann-Whitney U tests). All data were expressed as means ±SD. ***P < 0.001.

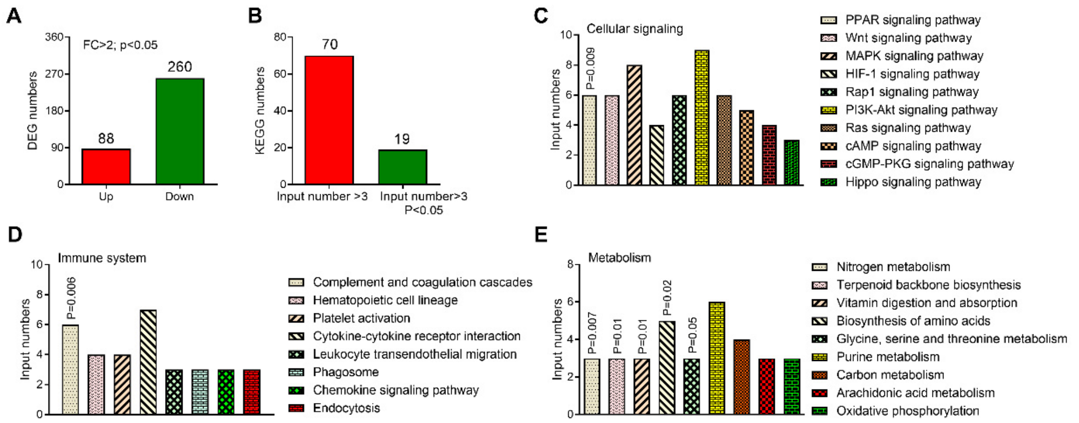

Supplementary Figure S2. The results of transcriptome analysis in L-serine / Control. A: The up/down-regulated DEGs in macrophages after 10 mM L-serine treatment. **B:** Total KEGG number and significant KEGG number in macrophages after 10 mM L-serine treatment. **C:** Pathways associated with cellular signaling in transcriptome sequencing. **D:** Pathways in transcriptome sequencing that is associated with the immune system. **E:** Metabolic pathways in transcriptome sequencing.

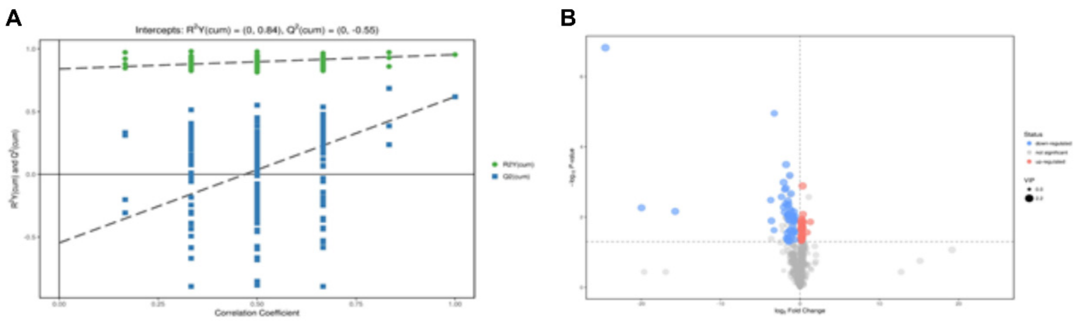

Supplementary Figure S3. The results of metabolome analysis in L-serine / Control. A: OPLS-DA permutation plot. **B:** The volcano plot of differentially expressed metabolites (Each point represents one individual. Blue point means: down-regulated; Red point means: up-regulated; Grey point means: not significant).
