# Supplementary material for: Exogenous L-Serine Alleviates Pasteurella multocida-Induced Inflammation by Reprogramming the Transcription and Metabolism of Macrophages
Source: Vet Sci. 2025 Mar 7;12(3):254. doi: 10.3390/vetsci12030254 (PMC11945856; doi:10.3390/vetsci12030254)

Original Images for BlotsGellts in Figure 3A

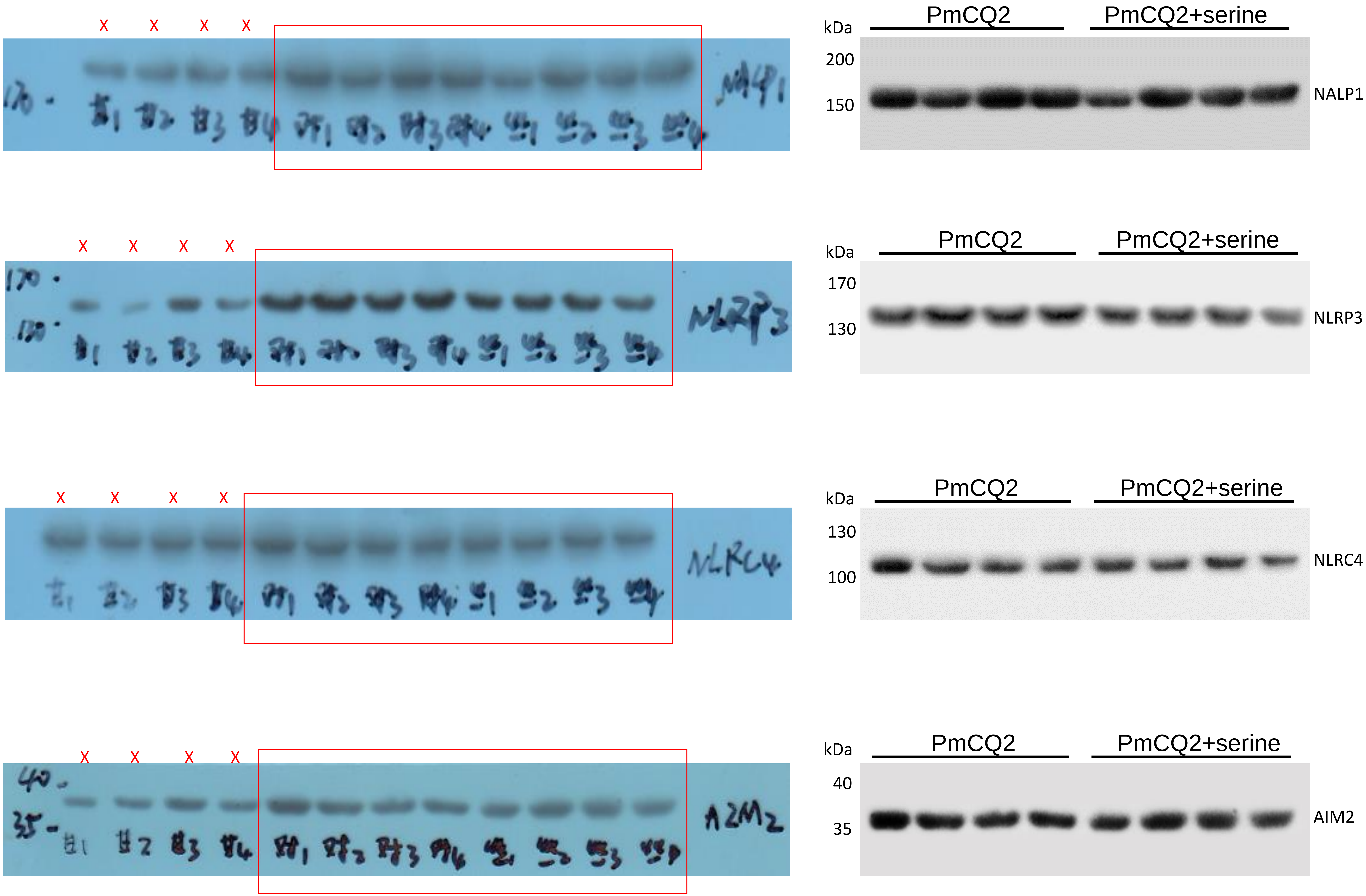

Original Images for BlotsGellts in Figure 3A

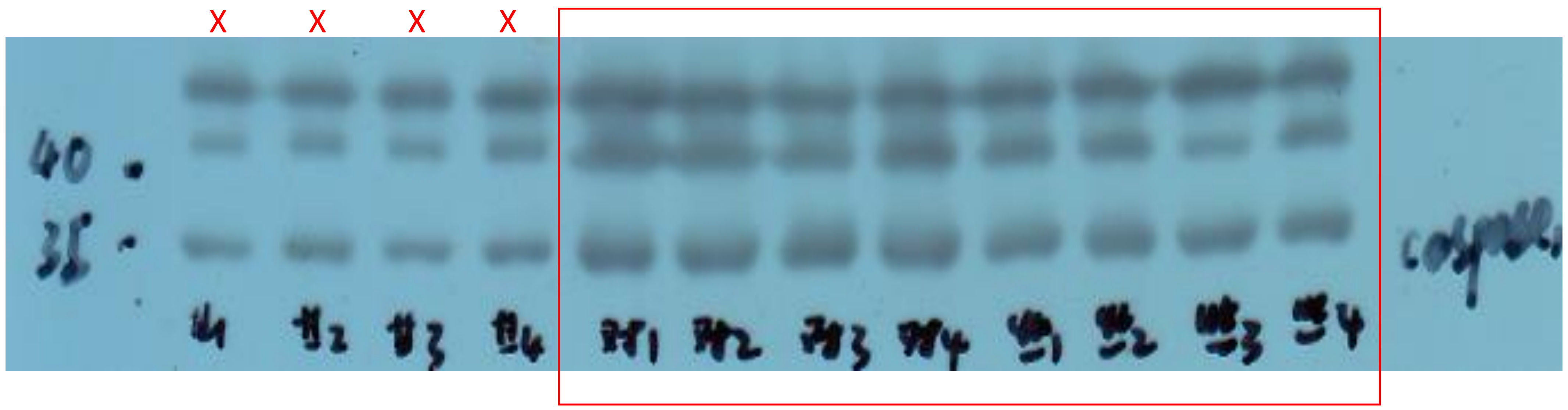

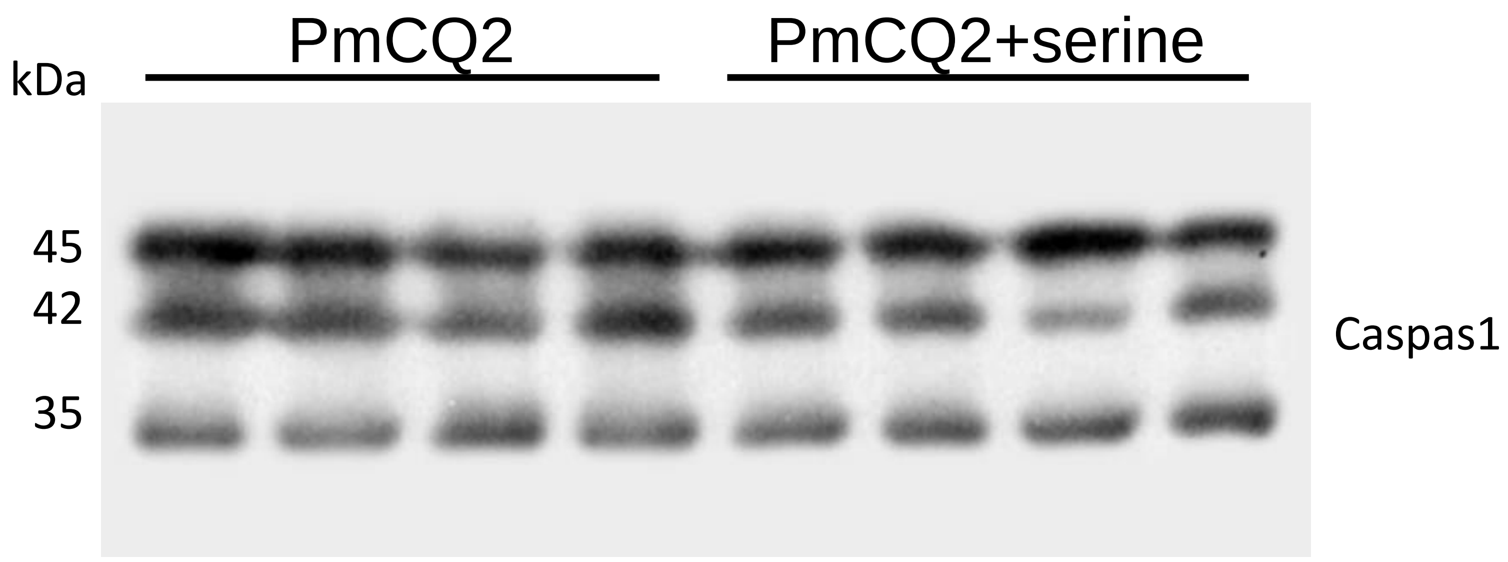

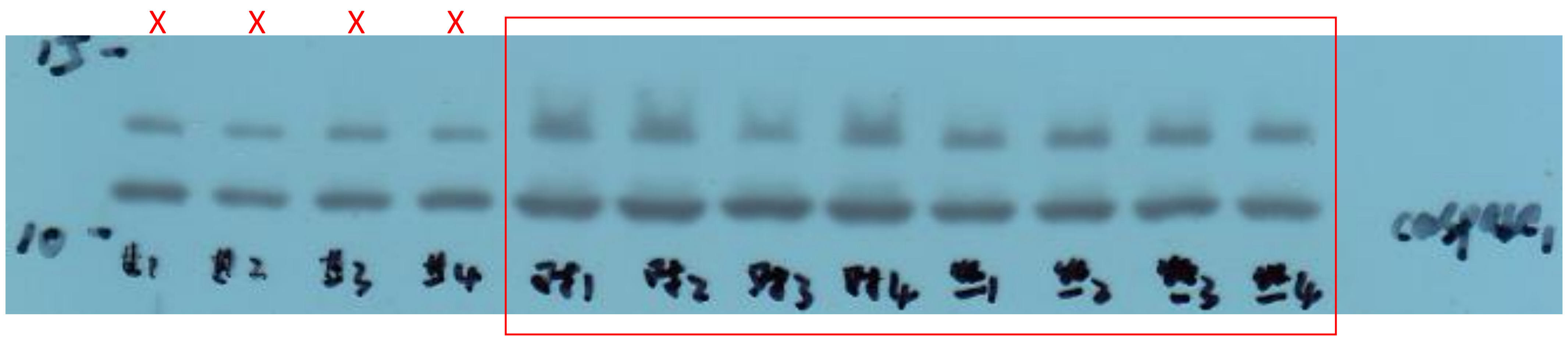

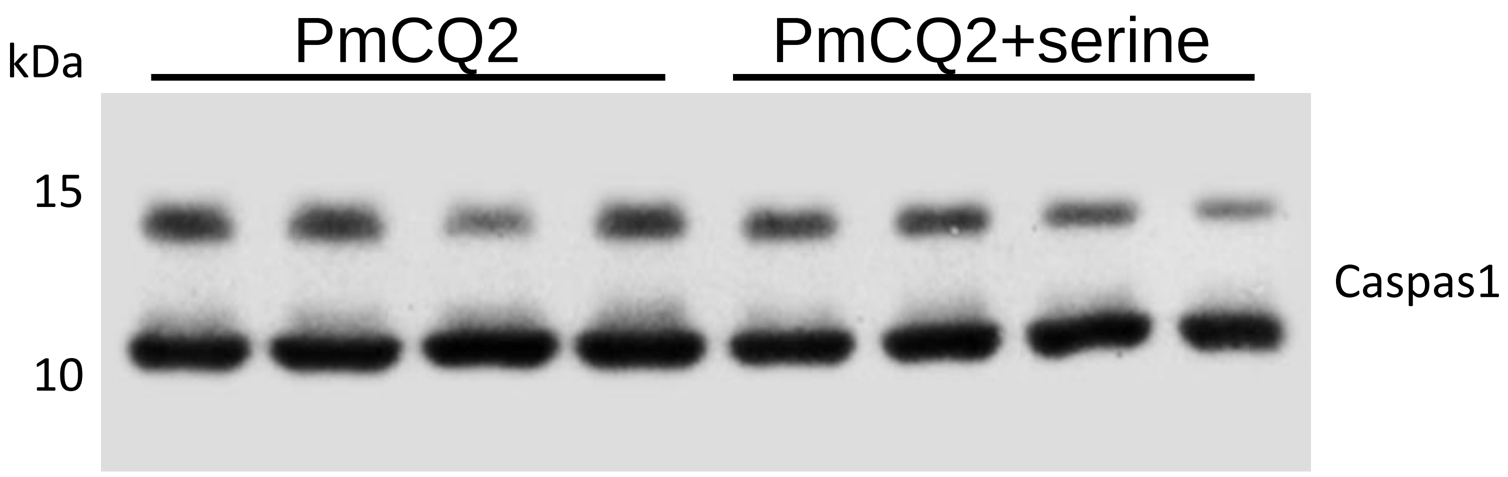

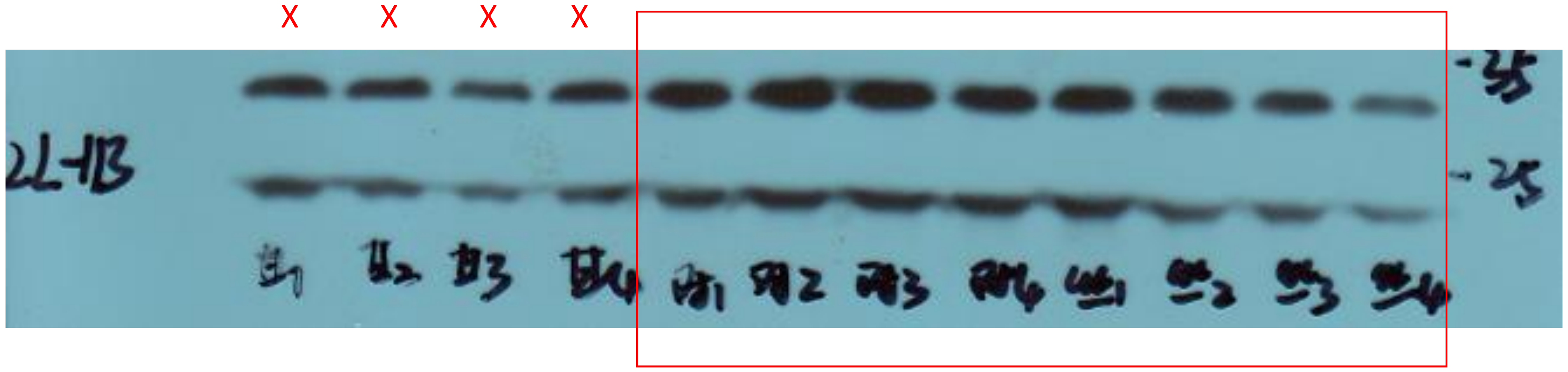

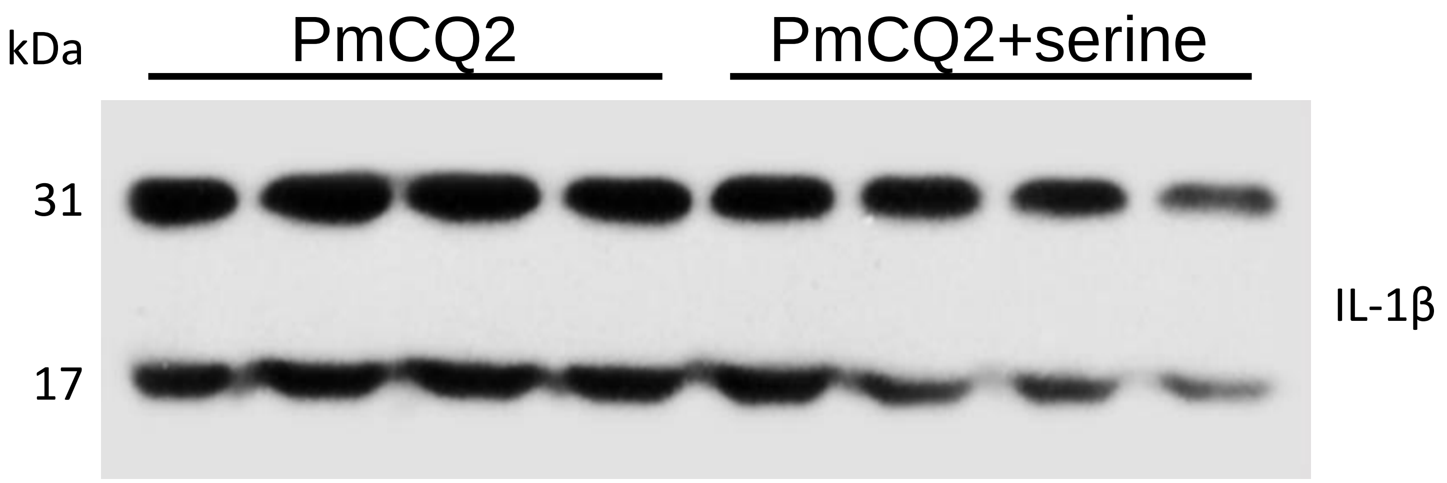

Original Images for BlotsGellts in Figure 3A

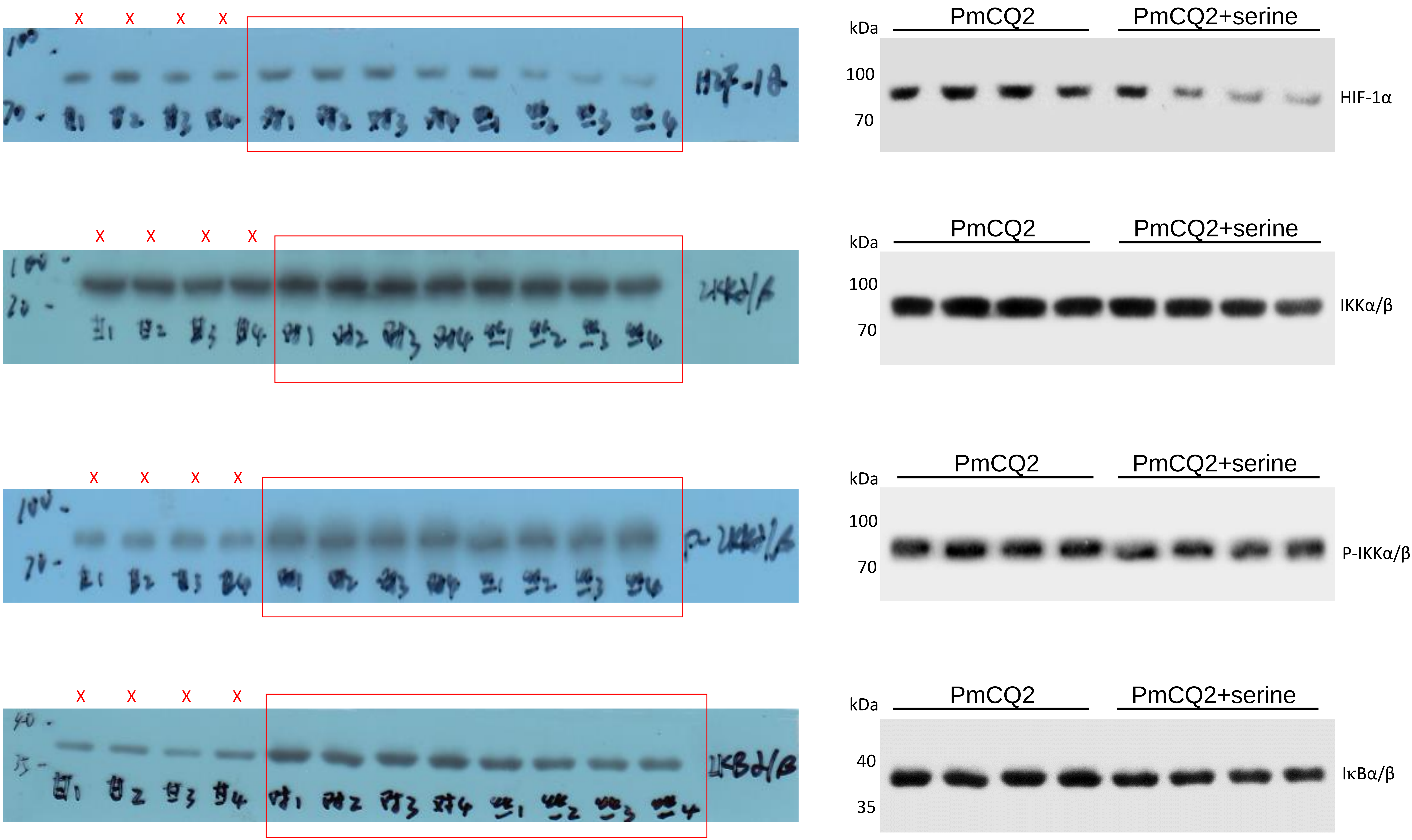

Original Images for BlotsGellts in Figure 3A

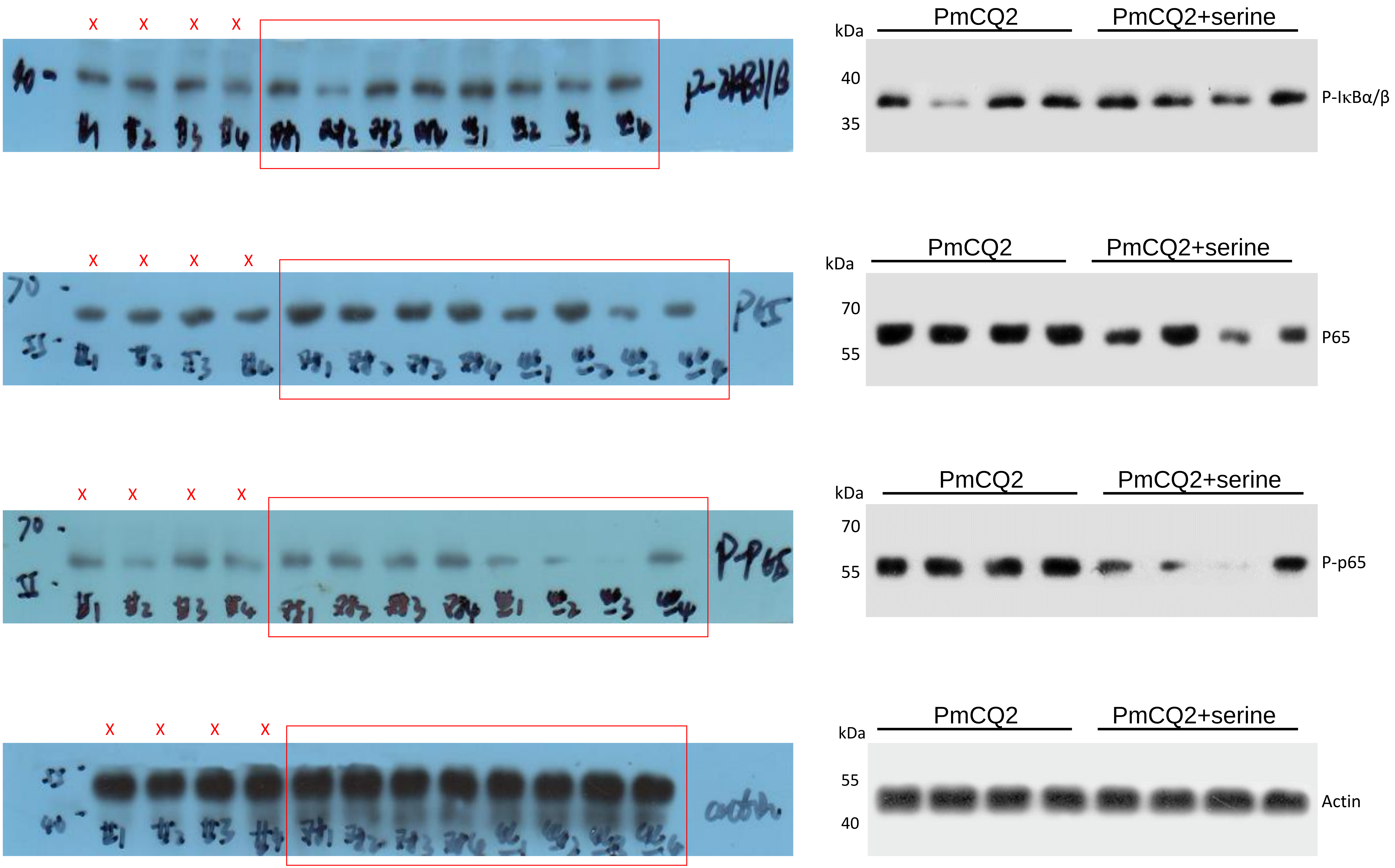

Supplement: Supplementary file 1 [file vetsci-12-00254-s001.zip › vetsci-3457843-WB figure.pdf]
